# Impact Strengthening of Laminated Kevlar/Epoxy Composites by Nanoparticle Reinforcement

**DOI:** 10.3390/polym12122814

**Published:** 2020-11-27

**Authors:** Abdel Hamid I. Mourad, Nizamudeen Cherupurakal, Farrukh Hafeez, Imad Barsoum, Farah A. Genena, Mouza S. Al Mansoori, Lamia A. Al Marzooqi

**Affiliations:** 1 Mechanical Engineering Department, College of Engineering, United Arab Emirate University, Al Ain 15551, UAE; 201890121@uaeu.ac.ae (N.C.); 201350166@uaeu.ac.ae (F.A.G.); 201303197@uaeu.ac.ae (M.S.A.M.); 201300729@uaeu.ac.ae (L.A.A.M.); 2 Mechanical Design Department, Faculty of Engineering, Helwan University, Cairo 11795, Egypt; 3 Department of Mechanical Engineering, University of Birmingham, Dubai 341799, UAE; f.hafeez@bham.ac.uk; 4 Department of Mechanical Engineering, Khalifa University, Abu Dhabi 127788, UAE; imad.barsoum@ku.ac.ae; 5 Department of Engineering Mechanics, Royal Institute of Technology-KTH, 10044 Stockholm, Sweden

**Keywords:** nanocomposites, Kevlar, epoxy, curing, thermo-mechanical properties

## Abstract

Herein, we report the fabrication and characterization of high-strength Kevlar epoxy composite sheets for structural application. This process includes optimization of the curing conditions of composite preparation, such as curing time and temperature, and the incorporation of nanofillers, such as aluminum oxide (Al_2_O_3_), silicon carbide (SiC), and multi-walled carbon nanotubes (MWCNT) in different weight percentages. Differential scanning calorimetry (DSC) was utilized to investigate the thermal stability and curing behavior of the epoxy, finding that a minimum of 5 min is required for complete curing under an optimized temperature of 170 °C. Moreover, mechanical characterization, including flexural and drop-weight tests, were performed and found to be in good agreement with the DSC results. Our results show that nanofiller incorporation improves the mechanical properties of Kevlar epoxy composites. Among the tested samples, 0.5% MWCNT incorporation obtained the highest mechanical strength.

## 1. Introduction

Fiber reinforced polymer (FRP) composites are more reliable and safer than traditional composites, such as concrete and other building materials. The advantages of FRP composites include dimensional stability and flexibility, low maintenance and user cost, ease of installation, handling, and longer service life. Compared to steel structures, FRP composition are lightweight, fire retardant, and able to withstand high temperatures [1,2,3,4,5,6]. Their production costs are also lower. In general, FRP compositions comprise the matrix (epoxy, polyurethane etc.) and fibers (glass fiber, carbon fiber, Kevlar etc.) as reinforcement [7,8,9,10,11].

Among the most commonly used fibers, Kevlar is well-known for its strength and heat resistance. Kevlar KM2 Plus (Aramid fiber) is an evolution of the original Kevlar fiber, formed by the condensation reaction between an amine (1, 4-phenylene-diamine) and an acid chloride (terephthaloyl chloride) [12]. Rigid spun Kevlar sheets are held together by hydrogen bonds, resulting in unique mechanical properties such as a high modulus of elasticity, high tensile strength, toughness, and thermal stability. KM^2+^ built by DuPont for military purposes provides the highest-grade protective fiber [13,14,15]. In addition to its unique mechanical properties, the chemical and corrosion resistance of Kevlar make it an excellent candidate for both offshore and onshore applications. At present, Kevlar is used offshore for high-pressure hydraulic hoses and to reinforce oil pipes that transport oil from the ocean to the production platform; in this role, it serves as a replacement for steel, which is susceptible to corrosion. Among onshore applications, it is suitable for small-diameter thermoplastic pipe reinforcement [16].

**Figure 1:**
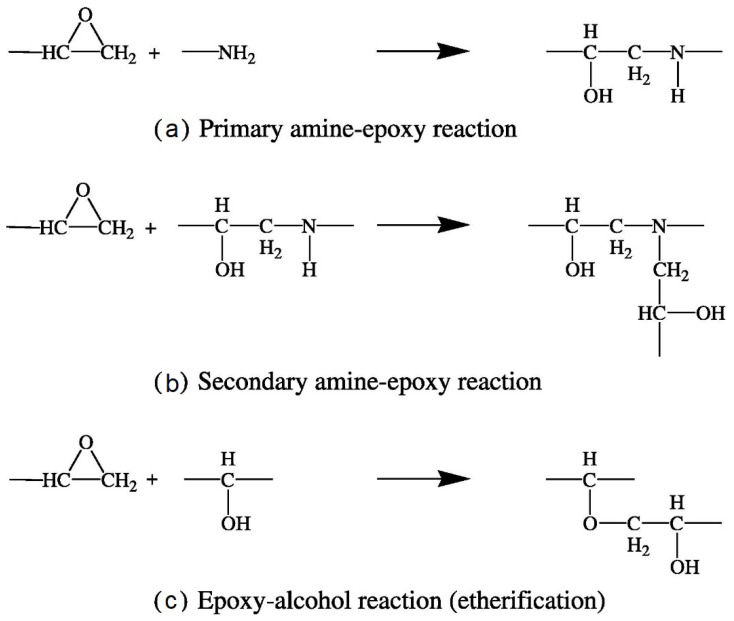
Mechanism of epoxy curing [17].

Epoxy resins are known for material properties such as heat resistance, chemical resistance, adhesiveness, and outstanding electrical properties. Their versatile applications include electrical insulation, adhesives, and coatings. Their curing requires either homopolymerization of the epoxy group or addition polymerization between the epoxy group and other reactive molecules. Hardeners are added to initiate and accelerate the curing process. During the curing reaction, liquid epoxy becomes a hard plastic through the formation of a three-dimensional crosslinked network. Curing progresses through chain growth polymerization, crosslinking by secondary amines, and crosslinking by tertiary amines. Figure 1 illustrates typical curing reactions between the oxirane group of the epoxy monomer and amine groups of the hardening agent [17].

Curing reactions are strongly dependent on time, temperature, and the pressure applied during the curing process. Studies have shown that increasing the curing temperature results in accelerated curing reactions in epoxy resins. Furthermore, the transition time of gelation and vitrification decreases with increased curing temperatures [18]. Additional investigation will be conducted during this research paper in order to determine optimal curing temperatures. It is important to observe the effect of curing temperature on epoxy resins since they are used in various crucial applications, such as coatings, adhesives, laminates, matrices for composite materials, and structural components, as well as applications that require chemical and heat resistance, specific mechanical properties, and electrical insulation. This versatility is due to low shrinkage rates during curing and the ability of these materials to withstand large temperature ranges, from −50 to +120 °C. The epoxy resin must be mixed with hardener (referred to as the “curing agent”) at a certain ratio before being cured, at which time it is valid for marketing either in its solid (crystalline/amorphous), liquid, or gaseous state and may be packed along with the epoxy glues required to harden the resin. Amines, imines, phenolics, and anhydrides are commonly used as hardeners [19].

Kevlar epoxy composites are widely and effectively used for military, aerospace, and automobile industry purposes, thus researchers have aimed to maximize the mechanical properties of the composite by adopting different synthesis and optimization procedures. The wet lay-up technique is found to be most inexpensive and convenient method of composition fabrication. Moreover, to enhance specific mechanical properties, nanofillers such as MWCNT and graphene have been utilized during the fabrication process. Sultan et al. [20] fabricated Kevlar epoxy composites using the simple wet lay-up technique and obtained enhanced mechanical properties including impact, tensile, and flexural strength greater than those of aluminum.

Vivekanandan et al. [21] studied the effect of nanoclays on the mechanical properties of Kevlar-glass fiber-epoxy composites, keeping the number of layers constant (14 layers) and adding 2 wt.% clay. Their results showed a slight increase in thermal stability up to 98 °C and an enhanced loss modulus in the temperature range 0–160 °C. Mehmet et al. [22] conducted a comparative study between Kevlar, carbon, and glass fiber reinforced epoxy composites. Silicon carbide (SiC) was incorporated in different weight percentages (0–20%) to enhance the tensile and impact properties of the studied composites, demonstrating that SiC was chemically compatible with all three. Moreover, the tensile and impact properties were found to be highly dependent both on the type of fiber and on the SiC content. SiC incorporation enhanced the mechanical properties of glass/epoxy composites but reduced that of carbon fiber/epoxy composites. No effect was seen in Kevlar/epoxy composites. In contrast, Derradji et al. [23] used SiC in phthalonitrile resin composites to show that mechanical strength tended to increase with increasing wt.% of SiC. In a similar study, Chakraborty et al. [24] used a polyurethane (PU)–epoxy mixture matrix and carbon nanofiber (CNF)-coated Kevlar fiber as fiber reinforcement. The CNF was coated via flame deposition and the composite was fabricated using the wet lay-up technique. The prepared composites (CNF-Kev/PU-Epoxy) obtained a fracture toughness of 11.7 MPa and a tensile strength of 139 MPa, with a maximum elongation of 47%.

Our study of Kevlar/epoxy composites mainly focused on their ballistic application. Due to the high strength to weight ratio of the Kevlar material, Kevlar/epoxy composites are used for body armor and aircraft structures. Moreover, different nanofillers may be incorporated to enhance their strength. Pekbey et al. [25] studied the ballistic impact behavior of Kevlar-filled epoxy matrices utilizing three different fillers (nanoclay, nanocalcite, and nanocarbon) at different wt.% values from 0% to 2%. Sample were fabricated via the wet lay-up technique. The addition of 2 wt.% nanocalcite and 1 wt.% nanoclay improved the ballistic strength of the composites; however, the incorporation of nanocarbon led to reduction in impact resistance. Micheli et al. [26] developed a hybrid composite using Kevlar and glass fiber that was able to shield against electromagnetic interferences (EMIs) while withstanding mechanical shocks. To improve its shock absorption properties, CNT was incorporated. The composite achieved 80 dB shielding and thus approaches metallic behavior. Measuring the impact strength using an inhouse impact tester showed that the composites were able to absorb high-energy impacts in spite of local delamination of the layered structure.

Recently, numerous companies have started to manufacture Kevlar with enhanced impact resistance. In Yang et al. [27], Auxetic Kevlar^®^ developed by the UMass Dartmouth fabric development lab was evaluated in terms of its impact performance and compared with Kevlar 3000 denier. Vacuum infusion was used to fabricate Kevlar/epoxy composites. The results show that auxetic Kevlar^®^ composites have significantly improved fracture toughness (up to 225% that of regular woven Kevlar^®^ composites). Furthermore, with flocking, a 577% enhancement in initiation toughness was found and the damaged area was minimal in Auxetic Kevlar^®^ compared to its woven counterpart, suggesting that it led to a rection in absorbed impact energy.

In the present work, a recently developed Kevlar, known as KM2 Plus, was utilized to fabricate Kevlar/epoxy composite sheets. KM2 Plus is the highest grade Kevlar fibers developed by Dupont specially for military purposes. The aim of this work is to optimize the curing parameters of this new composite and enhance its mechanical properties, including flexural and impact strength, via the incorporation of nanofillers such as such as SiC, Al_2_O_3_, and MWCNT.

## 2. Materials and Methods

### 2.1. Materials

Kevlar KM2Plus: 767 aramid or Kevlar^®^, KM2Plus 600 denier, a registered trademark of DuPont, is a one of the most recent types of Kevlar available in the market and used in this work. Kevlar^®^, KM2Plus 600 denier was purchased from JPS Composite Materials Corporation (Anderson, SC, USA).

Epoxy Resin and Hardener: Araldite^®^ AY105, a medium viscosity unmodified epoxy resin based on bisphenol-A, was used in this study. It has very good performance at moderate temperatures (>200 °C) and has excellent chemical, water, and humidity resistance. Araldite^®^, a registered trademark of Huntsman LLC (The Woodlands, TX, USA), was used to bind layers of Kevlar fabric together. Low-viscosity cycloaliphatic polyamine hardener HY 2962—Aradur 42—from Huntsman LLC (The Woodlands, TX, USA) was used as the hardener. It will provide very good color stability and good resistance to aqueous neutral and alkaline medium. The recommended resin to hardener ratio is 5:1.

Nanofillers: Silicon carbide, SiC, with 1200 Grit number (15.3 µm particle size), aluminum oxide (Al_2_O_3_) with 50 µm particle size from PANADYNE Company (Warminster, PA, USA), 1200 grit number (15.3 µm particle size), and multi-wall carbon nanotubes (MWCNT) were obtained from Nanolab (Newton, MA, USA) with a purity greater than 85 wt.%. SiC and Al_2_O_3_ nanofillers were added to the epoxy resin at weight percentages of 1, 2 and 3 wt.%. MWCNT nanofillers were added with weight percentages of 0.25, 0.5, and 1 wt.%. Nanocomposite samples at higher weight percentages were difficult to fabricate due to the increased viscosity of the epoxy/nanocomposite solution and the increasing sedimentation rate of the nanofiller [28].

### 2.2. Method

Kevlar fibers received in the form of a roll were cut into patches of 20 cm^2^. Aluminum plates were cut into squares of 25 cm^2^. Hardener and epoxy were used at a weight ratio of 1:5. For the preparation of nanofiller-incorporated composites, epoxy was first mixed with nanoadditives, such as Al_2_O_3_ and SiC, and MWCNT in the required amounts before the addition of the hardener. The mixture prepared needed to be very well-mixed using a wooden spatula and sonicated for 15 min to ensure its homogeneity. Hardener was added to the sonicated mixture for accelerated curing. Following preparation of each component, they were integrated to prepare samples. A layer of industrial wax was applied on the inner side of the Al plates in order to minimize the possibility of the epoxy composite sticking to them. Thereafter, a base layer of epoxy composite was applied, and the first sheet of Kevlar KM2 fiber was placed. A metallic roller was used to eliminate any voids and air between the Kevlar and epoxy. Another layer of composite was applied using a metallic wiper to ensure a uniform layer application throughout the fiber. This process was repeated until 10 layers of Kevlar were prepared. The sample was then completely packed in a using transparent sellotape to avoid epoxy splashing during curing. When packing, some clearance was left to allow for the bleeding out of excess epoxy. A double-plate vertical hot press was used to rapidly cure the composite. The curing parameters were defined on the hot press with a pressure of 18 kN. When the curing process was completed, the sample was taken out of the hot press and left to cool down before un-packing the final cured product.

Multiple samples were prepared using different parameters in order to identify the optimum curing conditions based on mechanical testing results. The parameters changed included the curing time (5, 10, 15, and 20 min), curing temperature (100, 150, 170 and 200 °C) and the concentration of nanofiller: Al_2_O_3_ (1%, 2% and 3%) and SiC (1%, 2% and 3%) and MWCNT (0.25%, 0.5%, 1%). Several tests were performed and, based on the results obtained, the optimum curing conditions and nanofiller contents were concluded.

### 2.3. Characterization

#### 2.3.1. Differential Scanning Calorimetry (DSC)

In DSC, the amount of heat absorbed or released is calculated from the difference in heat flow between the sample and the reference [29,30,31,32,33,34,35]. In the current study, DSC was used to obtain the relationship between curing time and temperature. To obtain the curing time, epoxy hardener was maintained within the DSC instrument for 30 min at different temperatures. Results were recorded as heat flow with respect to time. This test was carried out using a TA-Instruments DSC Q200 device (TA-Instruments, New Castle, DE, USA) with a sample weight of 5–10 mg. Samples were kept at temperatures between 80 and 200 °C.

#### 2.3.2. Scanning Electron Microscopy (SEM)

A JEOL-JSM 7610F SEM (JOEL Ltd., Tokyo, Japan) was employed to visualize the microstructure of the prepared composites. SEM images were captured at magnifications up to 10,000×. Surface roughness and nanoparticle distribution were analyzed from these images.

#### 2.3.3. Three-Point Bending Test

An MTS Universal Testing machine (MTS system corporation, Eden Prairie, MN, USA) was used to determine the flexural modulus and strength of laminated nano-composite specimens. Five samples of each type were tested for uniformity and repeatability. The test was conducted following the ASTM D790 standard [5,36,37,38,39,40]. For three-point bending, eight 1.7 cm strips were cut from each sample, and their flexural modulus (E_bend_) and flexural strength (σ_fM_) were calculated. A support span-to-depth ratio of 16:1 and a constant overhead speed of 5 mm/min was used. The specimen was deflected until, and beyond, the maximum load was reached.

#### 2.3.4. Impact Test

Low-velocity impact testing was conducted using an Instron drop-weight impact tower (Instron, Norwood, OH, USA) [41]. Reinforced panels were placed on a circular support with an internal diameter of 40 mm and impacted centrally by a carriage with a 20 mm diameter tip. The panels were supported without being clamped and the impacted from a height of 400 mm by a mass of 30.448 kg. The impact velocity was; thus, 2.80 m/s, resulting in an impact energy of 119.44 J, which perforated all samples. Three successful sets of samples were tested in each case. The impact force, impactor displacement, time, and velocity were recorded. Energy–displacement plots were obtained for all samples. The perforation energy was determined using the trapezium rule to determine the area under each curve. Very few impact studies have been conducted using Kevlar/epoxy [42,43].

## 3. Results and Discussion

**Figure 2:**
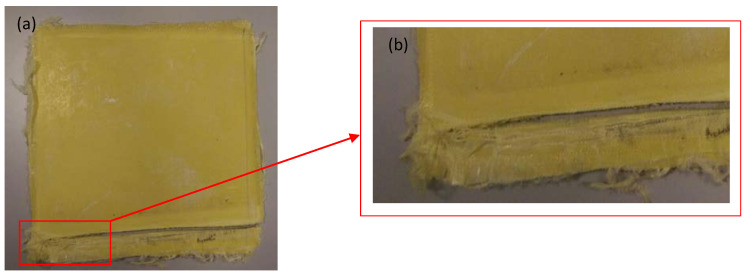
(**a**) Kevlar/epoxy composite plate prepared at 80 °C; (**b**) delamination of the Kevlar layers during cutting.

Samples were prepared using the wet lay-up technique, an example of which is shown in Figure 2a. Primary observations showed that samples prepared at lower temperatures (<100 °C) were delaminated, and their peeling off was observed during cutting (Figure 2b). This observation enabled us to set a lower temperature limit for the curing process and demonstrated that, when the temperature of curing is lower, Kevlar piles became very weakly bound, since the epoxy curing action to bond the sheets of Kevlar was weak.

### 3.1. Differential Scanning Calorimetry (DSC)

DSC results are summarized in Table 1. The curing time is determined as the time when graph attains study state. In the case of the 80 °C sample, it took more than 20 min for the curing to be more than 90% complete, whereas the 100 °C sample took only 15 min. Similarly, at temperatures of 170 and 200 °C, the composite sample took only 5 and 4 min, respectively, to cure. This confirms that curing time reduces with increasing temperature. However, mechanical characterization is required to choose optimal curing conditions. Both the TGA and DSC of the Kevlar composite were evaluated in earlier studies [44]. TGA results show that the decomposition temperature of the epoxy matrix is 360 °C, whereas Kevlar decomposes at 580 °C. DSC studies showed that the addition of nanofillers into epoxies improve the thermal stability of the composite.

### 3.2. Microstructure (SEM)

**Figure 3:**
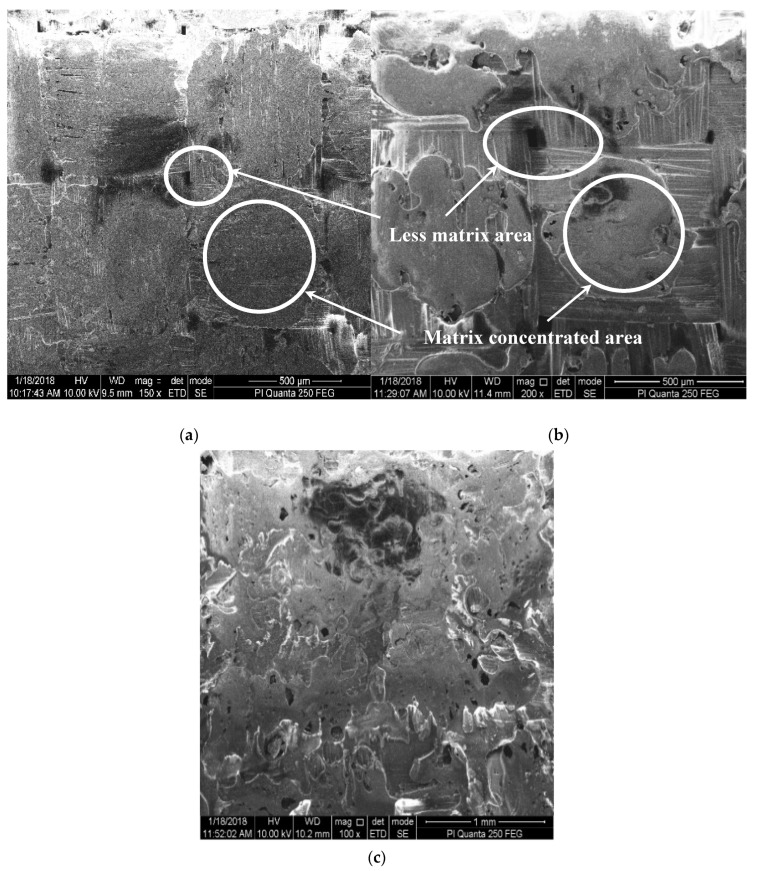
Texture of prepared composite sheets: (**a**) Control, (**b**) 1% Al_2_O_3_, (**c**) 1% SiC.

The prepared samples were characterized using SEM to analyze their textures and microstructure (Figure 3). In the control sample, the epoxy was found to be spread uniformly across the Kevlar surface; however, the corners of the woven fiber were found to have less epoxy (Figure 3a). For the 1% Al_2_O_3_-incorporated samples (see Figure 3b), the resin–nanofiller mixture was concentrated mostly toward the center rather than the edges whereas, in 1% SiC-incorporated samples (Figure 3c), it is present across the entirety of the Kevlar layers. The pattern remains the same as the concentration of nanofiller increases.

**Figure 4:**
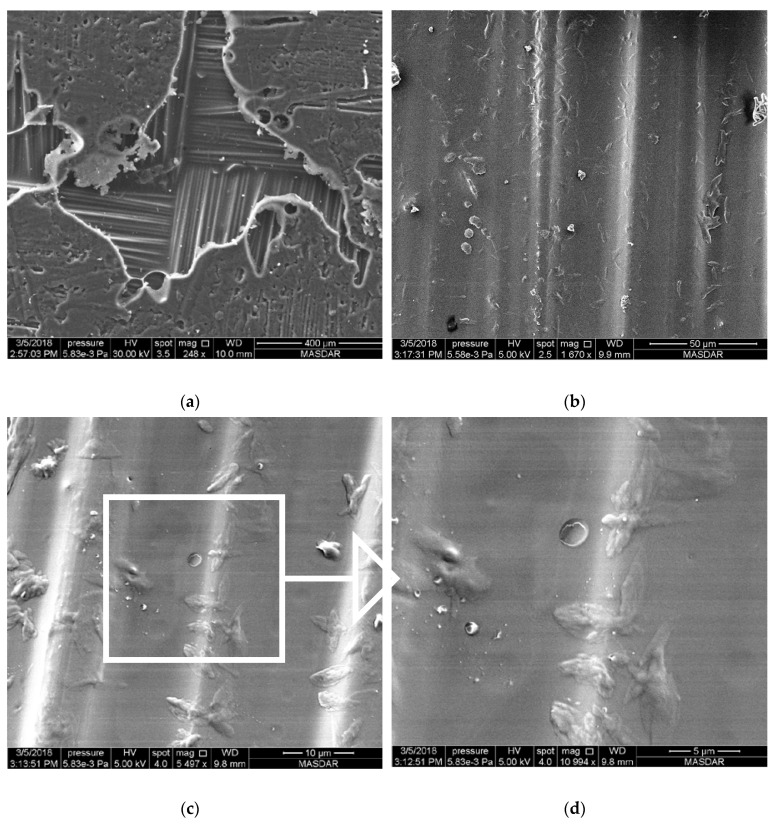
SEM image of Kevlar reinforced epoxy with 0.25% of carbon nanotubes at different magnifications. (**a**) 248×, (**b**) 1670× (**c**) 5497× (**d**) 10,994×.

**Figure 5:**
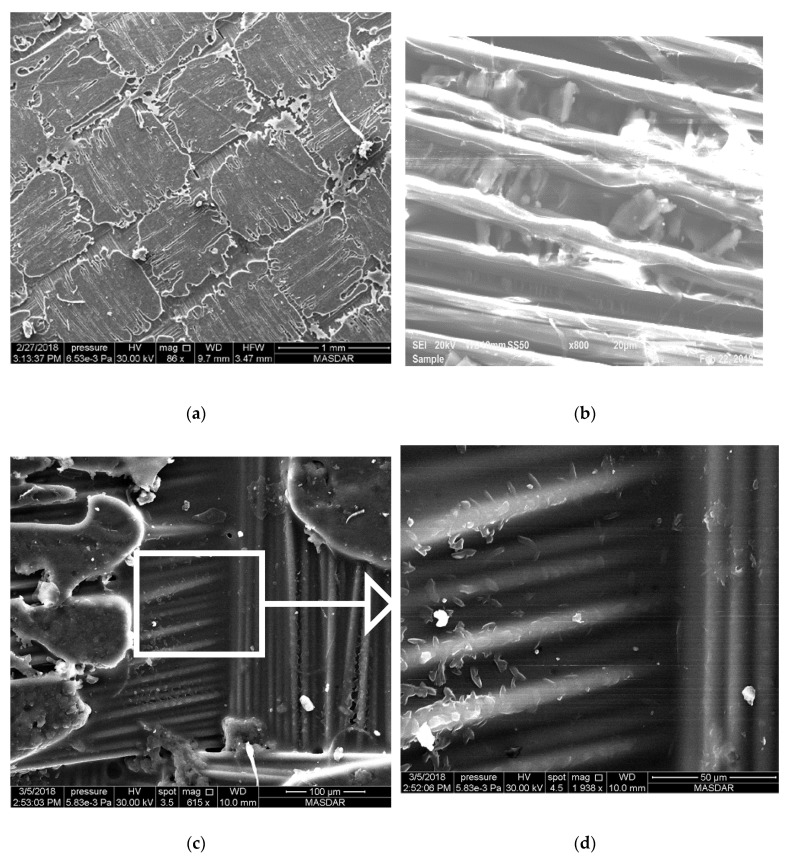
SEM image of Kevlar reinforced epoxy with 0.5% of carbon nanotubes at different magnifications. (**a**) 88×, (**b**) 8000× (**c**) 615× (**d**) 1938×.

**Figure 6:**
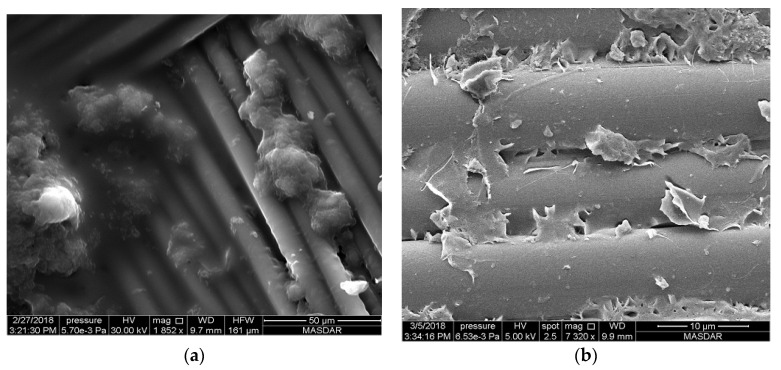
SEM image of Kevlar reinforced epoxy with 1.0% of carbon nanotubes at different magnifications. (**a**) 1852× (**b**) 7320×.

For MWCNT-incorporated samples, the pattern has a different appearance compared to the other two nanocomposite samples. Figure 4, Figure 5 and Figure 6 show images from samples containing 0.25%, 0.5%, and 1.0% MWCNTs, respectively. These images clearly show that carbon nanotubes aggregate irrespective of their percentage content in the composite. However, the distribution of agglomerated CNTs varies according to their overall percentage in the specimen. High-magnification SEM images allow the identification of individual Kevlar fibers with agglomerated CNTs. Figure 4a shows part of the sample in which the weave is exposed on the surface of a specimen containing 0.25% CNTs. Figure 4b–d show images of the same spot with different magnifications. Figure 4d is an inset of the enclosed area in Figure 4c, which shows that agglomerated CNTs are well-distributed in the area under focus. The same observation can be made over a larger area as shown in Figure 4b, where agglomerated CNTs are spread evenly across the whole area.

The distribution of the so-called aggregated CNTs is more prominent when the content in the composite increases to 0.5%. Figure 5d shows an inset of the enclosed square area in Figure 5c with higher magnification. CNTs can be seen stuck to and between fiber surfaces (see Figure 5b), well-distributed across the sample surface. It can thus be assumed that a similarly even distribution exists across all lamina of the composite. The overall surface of the composite with 0.5% CNT in Figure 5a is well-mixed and the distribution of fiber and epoxy with a weave pattern is clearly visible.

Increasing the CNT concentration to 1% substantially increases aggregation. Large localized concentrations and agglomerations of CNT covered with epoxy are found at various locations across the laminate surface. Figure 6 presents a representative instance with uniquely large size in order to explain the phenomena. In this feature, clearly exposed Kevlar fibers and large lump shaped substances are locally concentrated. It is possible that smaller units of aggregated CNTs are joined together with epoxy, further increasing the aggregation of CNT, thereby reducing the additional binding provided by CNTs in 0.5% concentration.

### 3.3. Three-Point Bending Test

**Figure 7:**
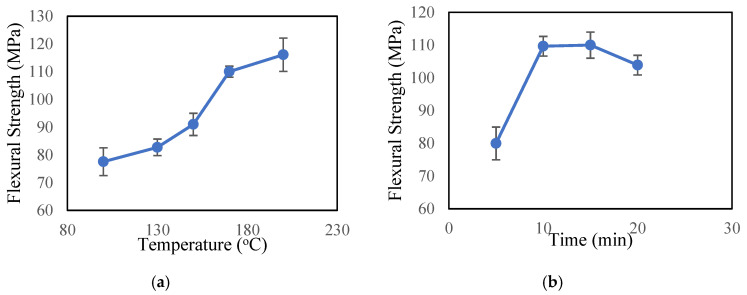
Flexural strength of control sample prepared at different curing conditions. (**a**) At constant time (15 min). (**b**) At constant temperature (170 °C).

Flexural strength and stiffness reflect the combined effects of the basic tensile, compressive, and shear properties of a material, whereas the flexural modulus represents resistance to bending deformation. To confirm the reproducibility of our results and demonstrate uniformity in sample preparation, five specimens from each sample were cut and tested using MTS. The load with respect to deflection curves was obtained using flexural test data. Figure 7 shows the results of flexural tests of composite samples (control) prepared at different curing conditions. Although curing time and temperature are optimized for the epoxy (DSC test), optimum conditions for the composite may vary slightly due to the incorporation of Kevlar. From Figure 7a, it can be observed that increasing curing temperature corresponds to increased flexural strength. From Figure 7b, the time required to cure the 170 °C sample can be seen between 10–15 min.

**Figure 8:**
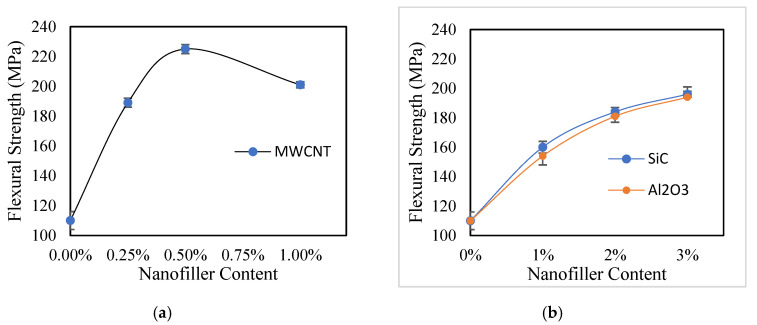
Flexural strength of nanofiller reinforced composite samples: (**a**) MWCNT, (**b**) SiC, and Al_2_O_3._

Optimized parameters were used to prepare nanofiller-incorporated samples, and a flexural test was conducted to observe variations in mechanical performance. The results show that the control sample has the lowest flexural strength (σFM) compared with the nanofiller-incorporated samples (Figure 8). In addition, as filler content increases, an increase in flexural properties is also observed. This arises from properties such as higher load-sharing capacity and heat distribution acquired by the composite following the incorporation of nanofillers [23,45]. Similarly, the large surface area of nanoparticles will enhance bonding and interactions between the fiber and the matrix. Samples with 0.5% MWCNT showed maximum flexural strength among all prepared samples. This can be interpreted to arise from the formation of a bridged network around the woven Kevlar fibers observed in microscopic images [46]. Furthermore, CNTs form dangling bonds within the epoxy matrix, thereby strengthening CNT relative to SiC and Al_2_O_3_ and leading to the highest values of flexural strength (σ_FM_) and modulus (E_bend_) among all samples [46,47]. Furthermore, increasing CNT concentration above 0.5% inversely affected the strength of the composite, likely due to the carbon nanotube agglomeration observed in optical and electron microscopic images. This local agglomeration creates local high stress points, which could act as sources of failure [48,49,50,51,52].

### 3.4. Impact Test

**Figure 9:**
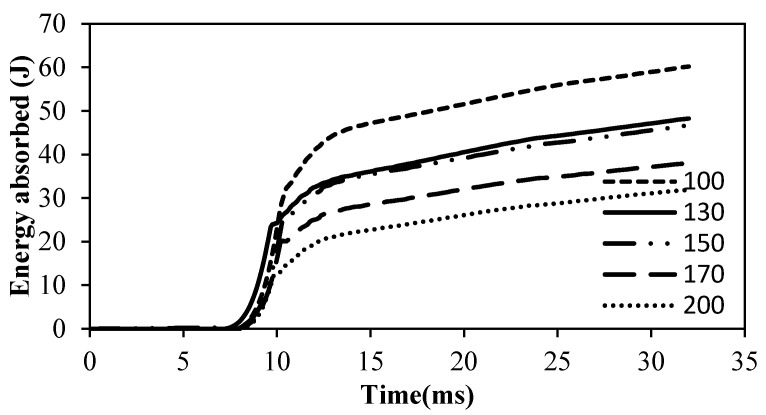
Impact test at constant curing time (15 min).

Figure 9 shows the energy absorbed by the Kevlar-reinforced epoxy samples during impact testing. The samples were prepared at different temperature conditions and the curing time was fixed at 15 min. It can be observed that the indenter hits the surface of the Kevlar plate at around 25 mm and the impact displacement starts increasing smoothly. Data collected prior are ignored since, at that time, the indenter is traveling and has not yet impacted the panel. This demonstrates that energy is being absorbed by the material as it deforms. At displacements between 25 and 35 mm, all of the Kevlar layers were broken, indicated by perforation through the plies. The experiment continued until the pile was completely perforated, at which point the graph shows a plateau. The prepared composite sample showed an increasing trend in the maximum absorbed energy when the curing temperature decreased. The sample cured at 100 °C had a maximum energy of 45 J, whereas for the 200 °C cured sample, the maximum absorbed energy was around 20 J. These observations reveal that samples with lower curing temperatures require more energy for perforation. This can be compared with the preliminary observations of this research showing that, at lower temperatures, the bonding between the Kevlar piles are very low. Hence, each Kevlar layer provides individual resistance to the impact load causing the perforation energy to increase. Samples cured at higher temperatures are bonded tightly, causing the entire composite resistance to act as single resistance, leading to rapid perforation through the sample.

**Figure 10:**
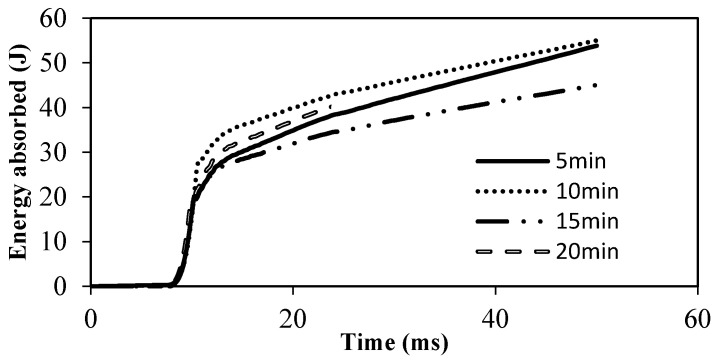
Impact test at constant curing temperature (170 °C).

Similarly, a test was conducted to evaluate changes in impact properties with respect to curing time at a fixed curing temperature of 170 °C. The energy absorbed with respect to displacement is shown in Figure 10. It is evident from Figure 10 that, for all samples, penetration started at an energy of 20 J. Additionally, perforation occurred at an average energy of 30 (±3) J in all samples. This confirms the observations of the DSC test, in which the sample 170 °C took only 5 min to cure when the sample was maintained under these conditions for a prolonged time. No significant effects on mechanical properties were observed, hence no changes could be seen in the samples cured for more than 5 min. We conclude that optimized parameters of 170 °C and 10 min give the maximum performance in all tests.

**Figure 11:**
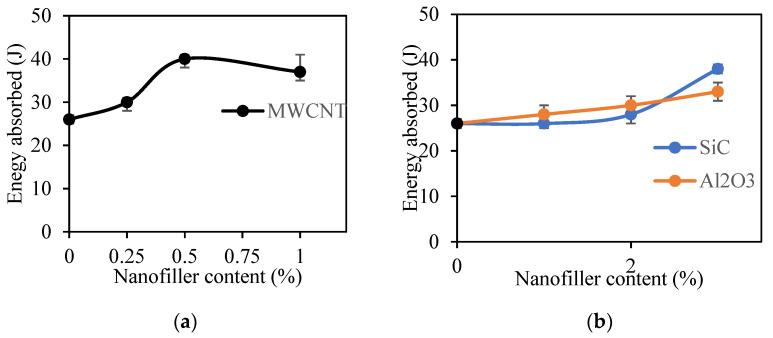
Impact test of nanofiller incorporated Kevlar/epoxy composites: (**a**) MWCNT, (**b**) SiC, and Al_2_O_3_.

Figure 11 compares the results obtained in nanofiller-reinforced specimens. For SiC-incorporated Kevlar composites, a drastic enhancement in impact properties was noticed when 3% SiC was incorporated. Compared to the optimized control sample without nanofillers, the enhancement is approximately 46%. In the case of Al_2_O_3_-incorporated samples, impact strength increased gradually with increasing percentage of Al_2_O_3_. For MWCNT-incorporated samples, the 0.5% and 1% MWCNT-incorporated samples were able to absorb a maximum energy of 44 J. However, due to agglomeration in the 1% MWCNT specimen, the results were not uniform and the mean value was reduced due to deviation. The findings of these impact studies corroborate the aforementioned flexural tests.

## 4. Conclusions

Advanced Kevlar/epoxy composites with nanofillers of Al_2_O_3_, SiC, and MWCNT were prepared via the by wet lay-up technique. DSC tests reveal the relationship between curing time and temperature. 170 °C was shown to be the optimum curing temperature for maximizing the mechanical properties of the produced composites. In addition, microscopic images show the pattern of nanofiller distribution over the Kevlar/epoxy matrix. Flexural test results prove that flexural properties are enhanced by increasing the nanofiller content; however, composites with 0.5% MWCNT showed maximum flexural performance, which reduced at higher MWCNT concentrations. This is due to higher local agglomeration. Impact tests confirmed that, at lower curing temperatures, impact resistance results mostly from weak bonding between Kevlar layers. Impact studies at different curing times demonstrated that curing time imparted no significant effects on mechanical properties once the composite sample was manufactured. The impact performance of all composites was enhanced by the incorporation of nanofillers. The maximum impact energy was absorbed by a 0.5% MWCNT-incorporated Kevlar/epoxy composite. Overall, this study indicates that improvements in the ballistic strength of Kevlar/epoxy composites may be achieved by utilizing nanofillers.

## Figures and Tables

**Table 1:** Curing temperature–time relation.

Curing Temperature (°C)	Required Time (Minutes)
80	25
100	19
130	13
150	10
170	5
200	4
